# Supplementary material for: Lysosome-related organelles promote stress and immune responses in C. elegans
Source: Commun Biol. 2023 Sep 13;6:936. doi: 10.1038/s42003-023-05246-7 (PMC10499889; doi:10.1038/s42003-023-05246-7)
Supplement: Supplementary file 2 — Supplementary Information [file 42003_2023_5246_MOESM2_ESM.pdf]

Lysosome-related organelles promote stress and immune defenses in *C. elegans*

Supplementary Information

Gábor Hajdú, Milán Somogyvári, Péter Csermely, Csaba Sőti*

Department of Molecular Biology, Semmelweis University, Budapest, Hungary

Content:
Supplementary Figures 1-8 and legends
Supplementary Tables 1-3

***Corresponding author.** Department of Molecular Biology, Semmelweis University, P.O.Box 2, Budapest, H-1428, Hungary. E-mail: soti.csaba@med.semmelweis-univ.hu

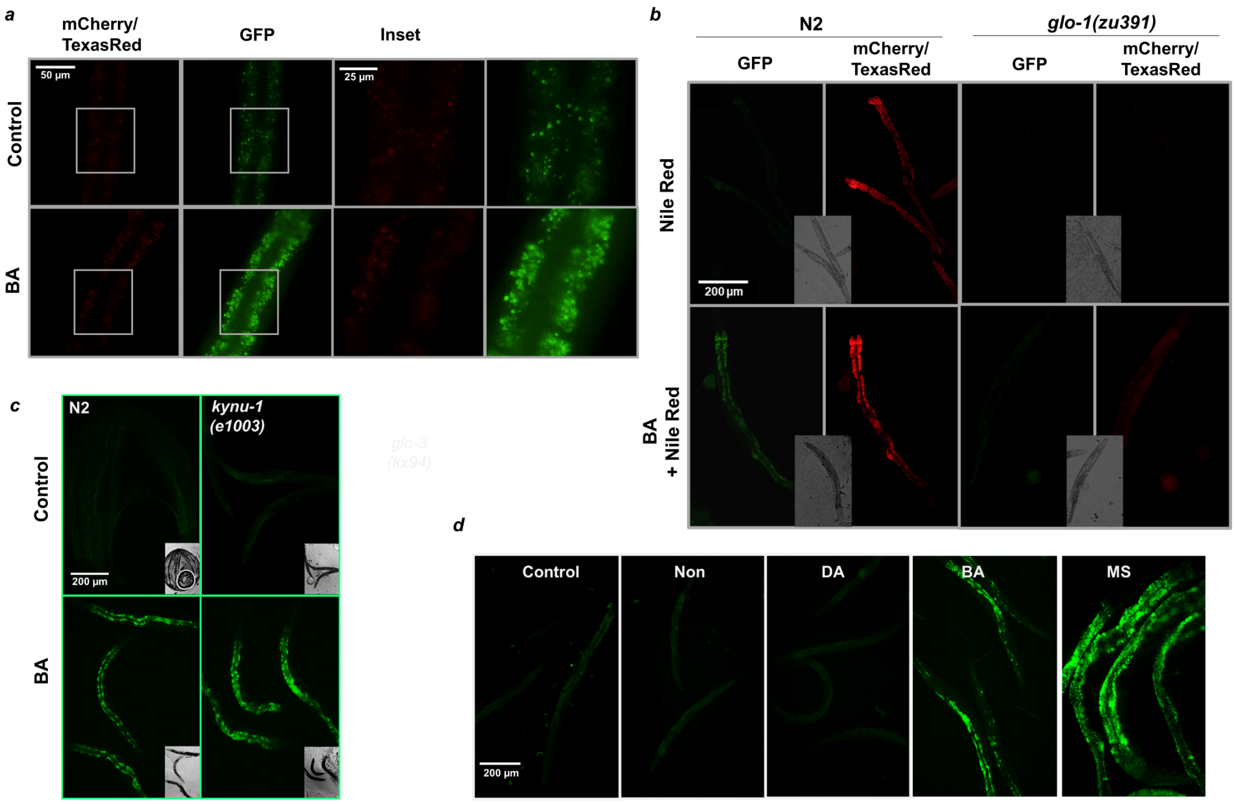

Supplementary Fig. 1. LRO-s accumulate an anthranilate-independent fluorescent material in response to BA and methyl-salicylate exposure, Related to Fig. 1. (a) Fluorescence microscopy images of BA-exposed wild-type worms in the mCherry and GFP channels. (b) Fluorescence microscopy images of control and BA-exposed N2 and *glo-1* worms simultaneously fed with Nile Red in the GFP and mCherry channels. (c) Fluorescence microscopy images of BA-exposed N2 and *kynu-1* mutants. (d) Fluorescence microscopy images of wild-type worms exposed to 2-nonanone (Non), diacetyl (DA), benzaldehyde (BA) and methyl salicylate (MS). Animals were exposed to 1 µl of undiluted odorants for 4-hours. Images represent 3 independent experiments with similar results.

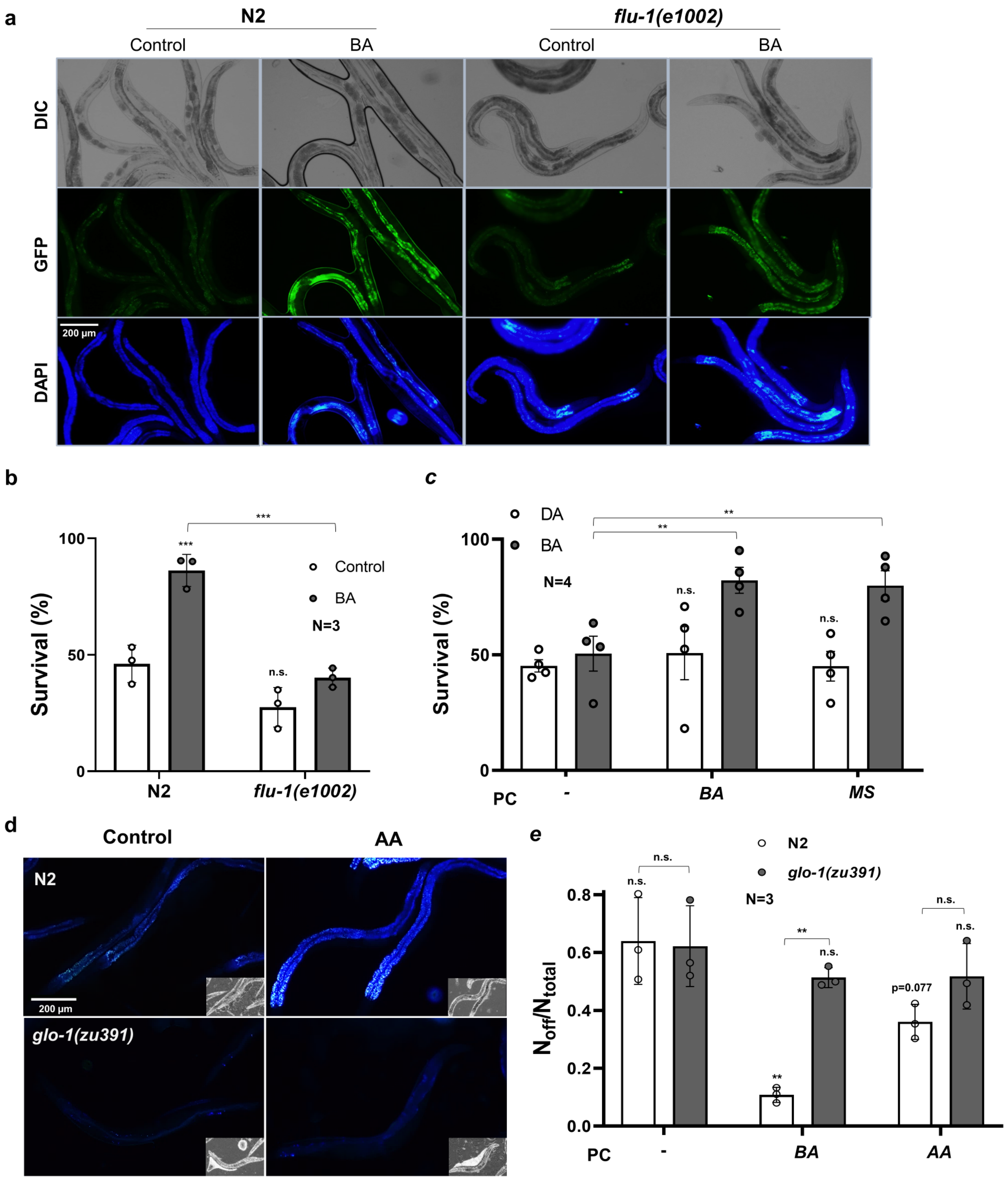

Supplementary Fig. 2. Effects of *flu-1,* methyl salicylate and anthranilate on adaptation to BA toxicity, Related to Fig. 2. (a) Fluorescence microscopy images of N2 and *flu-1* mutants upon BA exposure, assayed with GFP and DAPI filters. (b) Survival assays of control and BA preconditioned (PC) wild-type and *flu-1* worms exposed to a lethal dose of BA. (c) Survival assays of control, BA PC and MS PC animals exposed to a lethal dose of BA or diacetyl (DA). (d) Fluorescence microscopy images of N2 and *glo-1* mutants after overnight anthranilic acid (AA) treatment. (e) BA-induced food avoidance of naive, BA PC and AA PC

wild-type and *glo-1* mutants. Representative epifluorescence microscopy images were taken from at least two different experiments. Data are expressed as mean ± SEM. N, number of independent experiments. p values were obtained by two-way ANOVA with Fisher post hoc test. n.s., not significant; $*p < 0.05$, $**p < 0.01$; $***p < 0.001$.

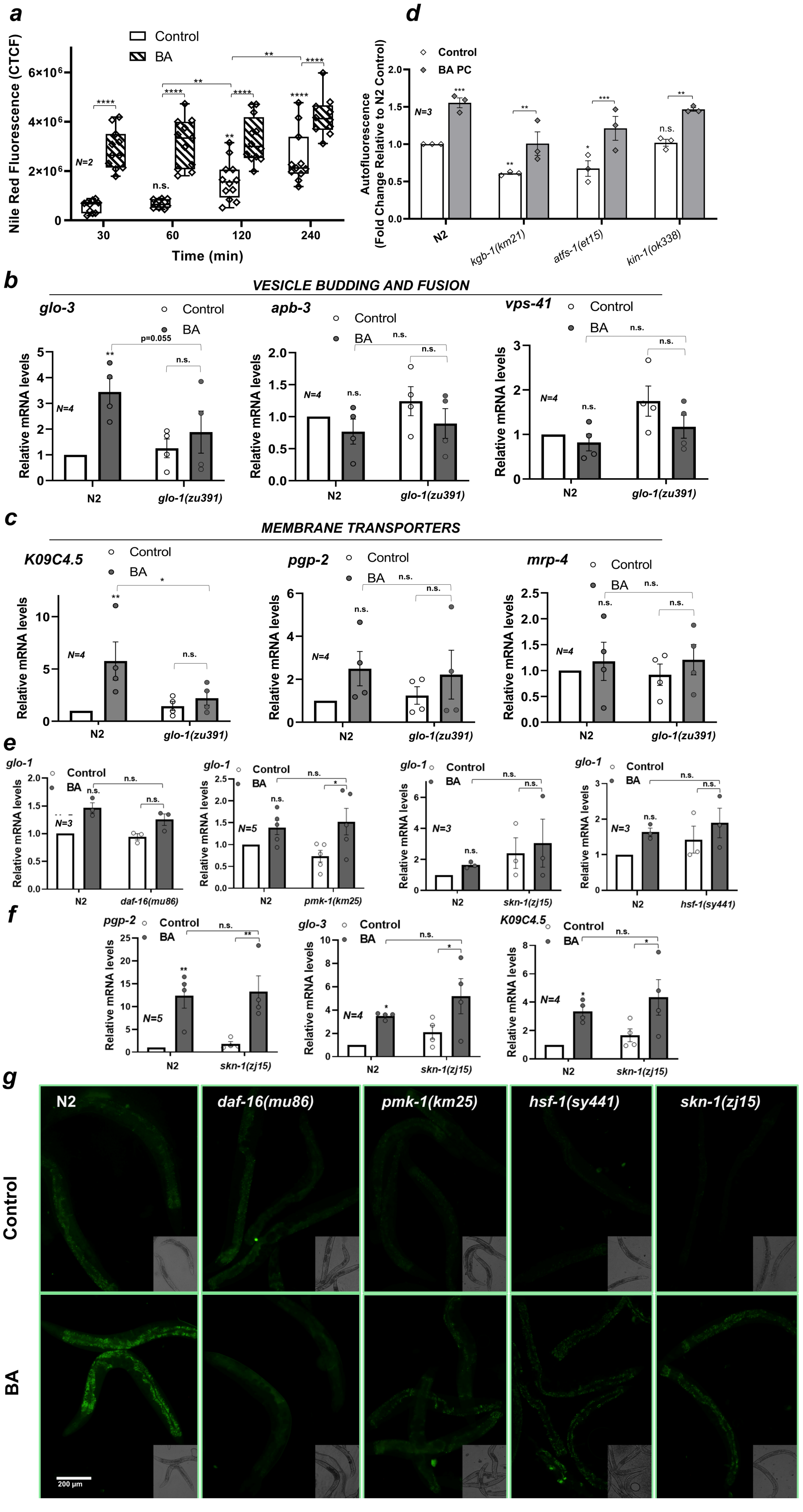

a
Control
BA
Nile Red Fluorescence (CTCF)
Time (min)
d
Control
BA PC
Autofluorescence (Fold Change Relative to N2 Control)
N2
kgb-1(km21)
atfs-1(et15)
kin-1(ok338)
b
VESICLE BUDDING AND FUSION
glo-3
apb-3
vps-41
Relative mRNA levels
glo-1(zu391)
c
MEMBRANE TRANSPORTERS
K09C4.5
pgp-2
mrp-4
e
glo-1
daf-16(mu86)
pmk-1(km25)
skn-1(zj15)
hsf-1(sy441)
f
pgp-2
glo-3
K09C4.5
g
N2
daf-16(mu86)
pmk-1(km25)
hsf-1(sy441)
skn-1(zj15)
Control
BA
200 μm

Supplementary Fig. 3. BA-induced, LRO-associated metabolic and transcriptional responses depend on diverse stress pathway regulators, Related to Fig. 3. (a) Quantification of the data from Fig. 3A. (b) Relative mRNA abundance of genes involved in LRO-related vesicle budding and fusion of N2 and *glo-1* mutants after a 24-hr BA treatment. (c) Relative mRNA abundance of LRO-related transmembrane transporters in N2 and *glo-1* mutants after a 24-hr BA treatment. (d) Relative autofluorescence of N2 and mutant nematodes after a 4-hr BA-treatment. (e) Relative abundance of *glo-1* mRNAs in N2 and mutant worms after a 2-hr BA treatment. (f) Relative mRNA abundance of BA-induced LRO-related genes in N2 and *skn-1* mutants after a 2-hr BA treatment. (g) Representative epifluorescence microscopy images of control and BA-exposed N2 and different mutant strains (corresponding to data in Fig. 3E). Boxes from panel A represent median and first and third quartiles and whiskers represent 10th to 90th percentiles. Other data are expressed as mean ± SEM. N, number of independent experiments each in triplicates. p values were obtained by two-way ANOVA in case of panels a and d and by one-way ANOVA in case of qRT-PCR measurements, with Fisher's LSD post hoc test following the evaluation of normal distribution significance by the Kolmogorov-Smirnov test (f). n.s., not significant; $*p < 0.05$, $**p < 0.01$, $****p < 0.0001$.

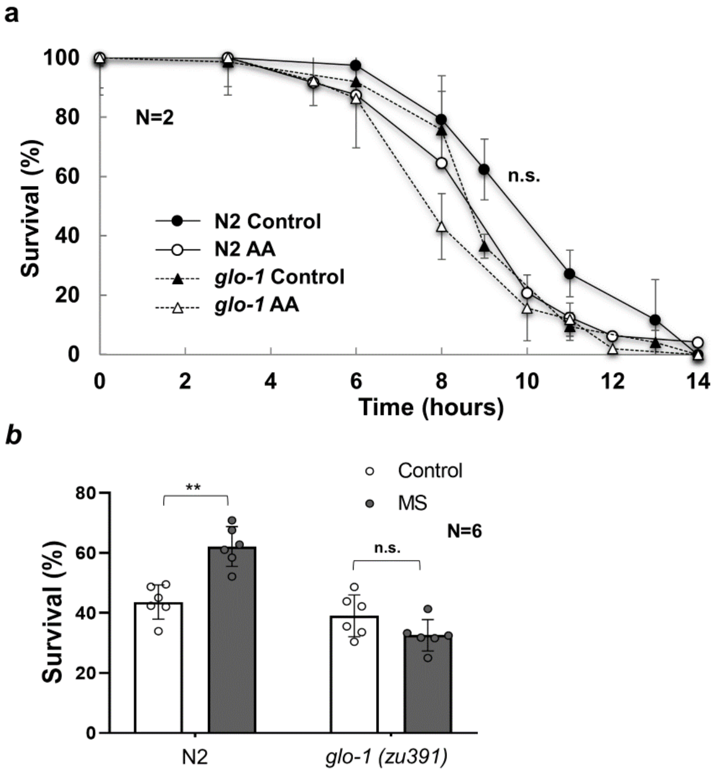

Supplementary Fig. 4. Anthranilate does not increase thermotolerance, methyl salicylate promotes paraquat tolerance, Related to Fig. 4. (a) Thermotolerance assays of N2 and *glo-1* animals preconditioned (PC) by AA. AA PC neither affects the survival of wild-type nor that of *glo-1(zu391)* mutants. Overall data are plotted from two independent assays. Detailed statistics are given in Supplementary Table S1. (b) Paraquat tolerance assays of N2 and *glo-1* animals preconditioned by MS. Data are expressed as mean ± SEM. N, number of independent experiments each in triplicates. p values were obtained by two-way ANOVA with Fisher's LSD post hoc test. n.s. not significant; **$p < 0.01$.

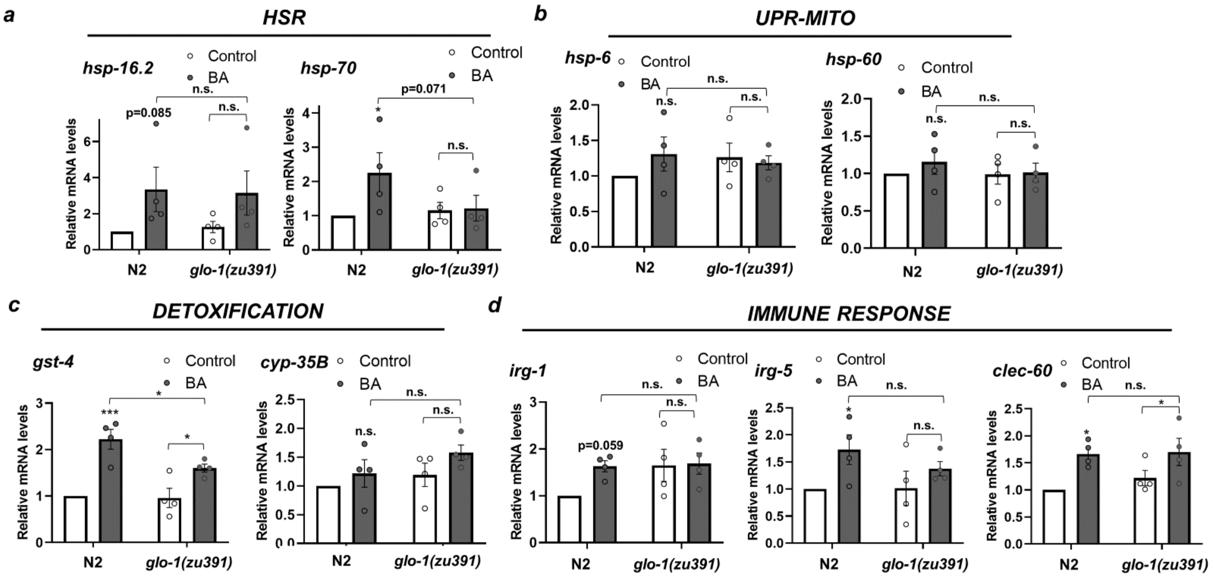

Supplementary Fig. 5. **BA-induced early expression of stress-, detoxification and immune responses in wild-type and *glo-1* worms, Related to Fig. 5.** The diagrams show the relative mRNA abundance of genes representing the following defense responses in wild-type and *glo-1* nematodes after a 2-hr BA treatment: (a) cytoplasmic heat shock response (HSR), (b) mitochondrial unfolded protein response (UPR-MITO) (c) detoxification enzymes and (d) pathogen recognition and defenses. N=4, number of independent experiments, each in triplicates. p values were obtained by two-way ANOVA with Fisher's LSD post hoc test. n.s., not significant; *$p < 0.05$; **$p < 0.01$; ***$p < 0.001$.

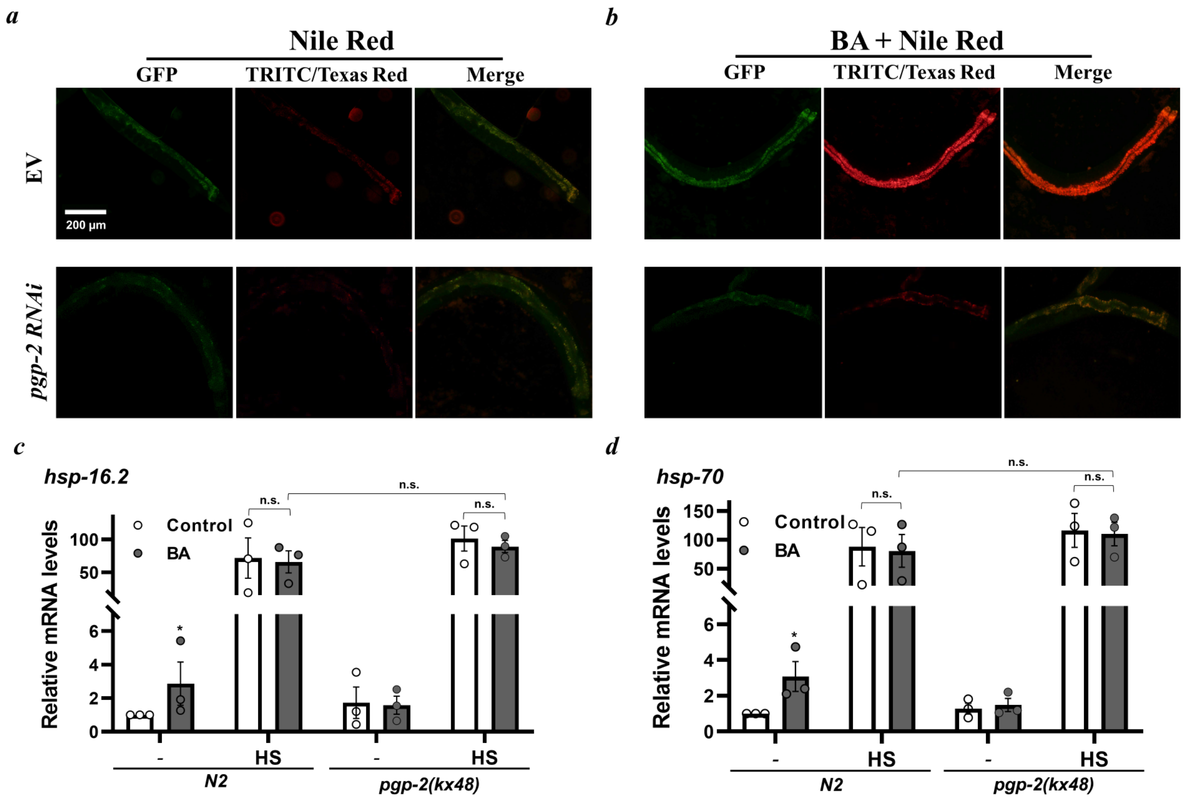

Supplementary Fig. 6. Loss of *pgp-2* prevents BA-induced autofluorescence and Nile Red accumulation, but does not affect the induction of the heat shock response, Related to Fig. 6. (a-b) Fluorescence microscopy images taken at different time points of Nile Red-fed EV and *pgp-2* RNAi-fed animals after a 4-hr vehicle and BA-treatment. Scale bar for panel b is the same as that for panel a. (c-d) Relative abundance of *hsp-16.2* and *hsp-70* mRNAs after a 2-hr heat stress at 31ºC, preceded by a 4-hr vehicle or BA preconditioning treatment. Representative epifluorescence microscopy images were taken from three independent experiments. Data are expressed as mean ± SEM. N=3, each in triplicates. p values were obtained by two-way ANOVA with Fisher's LSD post hoc test. n.s., not significant; $*p < 0.05$.

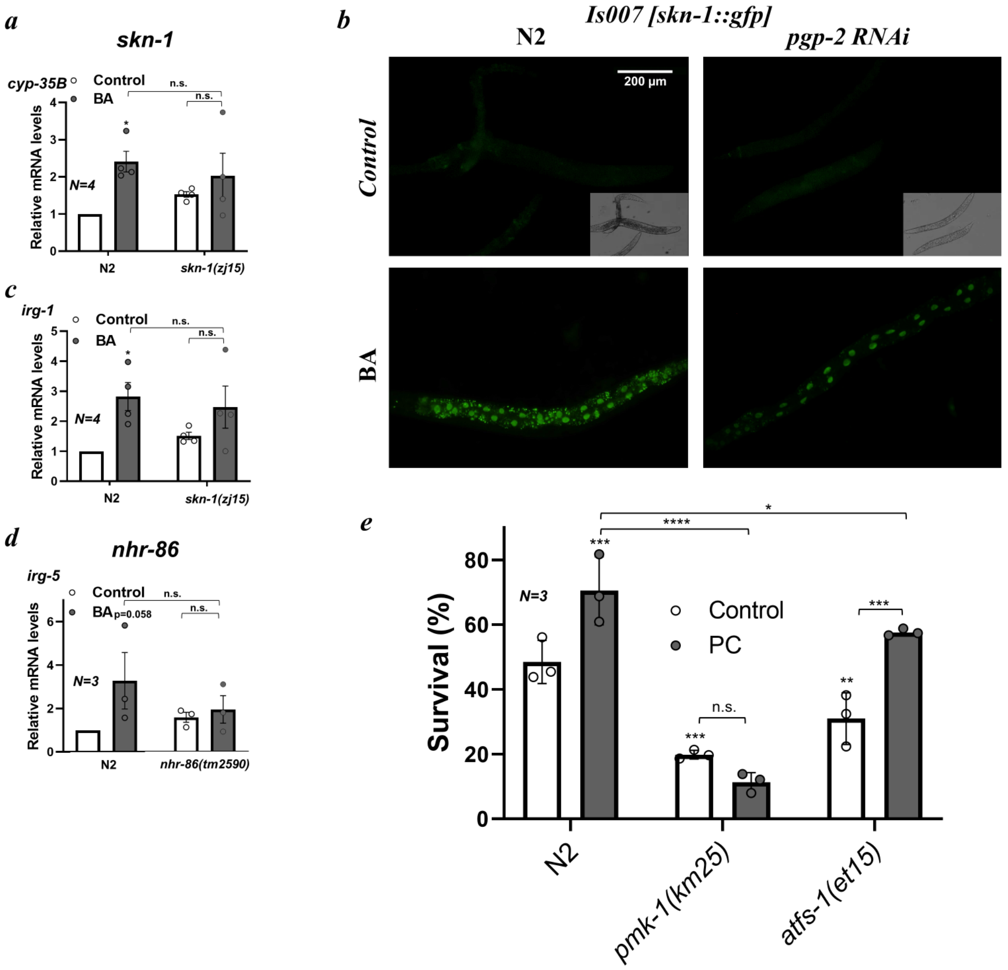

Supplementary Fig. 7. BA-activated LRO-dependent and -independent specific stress responses do not establish promiscuous crosstalks, Related to Fig. 7. (a) Relative abundance of the *cyp-35B* mRNA in N2 and *skn*-1 mutant nematodes after a 24-hr BA treatment. (b) Representative epifluorescence microscopic images of SKN-1::GFP nuclear translocation in EV and *pgp-2* RNAi-fed animals after a 30 min BA treatment. Please note the specific fluorescence of the larger nuclei and the granular LRO autofluorescence present only in EV-fed worms. (c) Relative abundance of the *irg-1* mRNA in N2 and *skn*-1 mutant nematodes after a 24-hr BA treatment. (d) Relative abundance of the *irg*-*5* mRNA in N2 and *nhr-86* mutant nematodes after a 24-hr BA treatment. (e) Paraquat toxicity assays of N2, *pmk-1* and *atfs-1* animals in 20 mg/ml PQ solution for 16 hours, with or without BA-preconditioning (PC). Representative epifluorescence microscopy images were taken from

three independent experiments. Data are expressed as mean ± SEM. N, number of independent experiments each in triplicates. p values were obtained by two-way ANOVA in case of qRT PCR measurements and one-way ANOVA in case of paraquat toxicity assays, with Fisher's LSD post hoc test. n.s., not significant; *p < 0.05; **p < 0.01; ***p < 0.001, ****p < 0.0001.

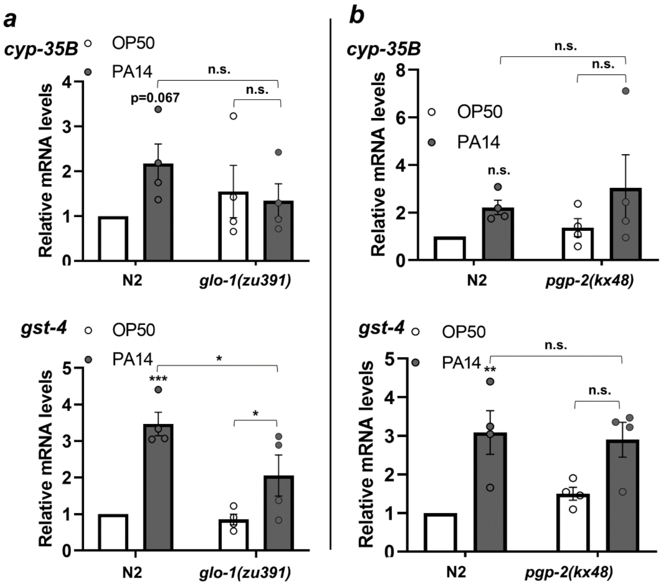

Supplementary Fig. 8. PA14 infection stimulates host detoxification enzymes, Related to Fig. 8. (a, b) Relative mRNA abundance of the detoxification enzyme genes *cyp-35B* and *gst-4* in wild-type, *glo-1* (a) and *pgp-2* (b) animals after a 16-hr PA14 exposure. N=4, number of independent experiments. p values were obtained by two-way ANOVA with Fisher's LSD post hoc test. n.s., not significant; *p < 0.05; **p < 0.01; ***p < 0.001.

Supplementary Table S1. Thermotolerance assays

Fig. 4a BA preconditioning

		Control		BA PC						Control				BA PC			
Assay No.	Genotype	n	Mean survival (hours)	n	Mean survival (hours)	% change *vs.* N2 Control	P value *vs.* N2 Control		Genotype	n	Mean survival (hours)	% change *vs.* N2 Control	P value *vs.* N2 Control	n	Mean survival (hours)	% change *vs.* *glo-1* Control	P value *vs.* *glo-1* Control
1	wild-type N2	94	11.32±0.43	103	12.10±0.49	107	n.s.		*glo-1(zu391)*	91	10.29±0.46	91	n.s.	85	9.85±0.40	96	n.s.
2	wild-type N2	103	9.38±0.36	126	10.80±0.23	115	<0.01		*glo-1(zu391)*	97	9.00±0.38	96	n.s.	97	9.97±0.34	111	n.s.
3	wild-type N2	106	8.64±0.36	110	9.60±0.39	111	n.s.		*glo-1(zu391)*	100	7.70±0.25	89	<0.05	94	7.27±0.24	94	n.s.
4	wild-type N2	91	7.74±0.34	106	9.313±0.58	120	<0.001		*glo-1(zu391)*	85	8.68±0.61	112	p=0.01	94	7.61±0.36	88	<0.01
5	wild-type N2	85	8.08±0.33	91	10.44±0.42	129	<0.001		*glo-1(zu391)*	59	7.85±0.44	97	n.s.	89	7.48±0.34	95	n.s.
**Overall**	**wild-type N2**	**479**	**9.03±1.42**	**536**	**10.45±1.10**	**116**	**<0.01**		***glo-1(zu391)***	**432**	**8.70±1.04**	**96**	**n.s.**	**459**	**8.43±1.35**	**97**	**n.s.**

Fig. 4b DA preconditioning

		Control		DA PC						Control				DA PC			
Assay No.	Genotype	n	Mean survival (hours)	n	Mean survival (hours)	% change *vs.* N2 Control	P value *vs.* N2 Control		Genotype	n	Mean survival (hours)	% change *vs.* N2 Control	P value *vs.* N2 Control	n	Mean survival (hours)	% change *vs.* *glo-1* Control	P value *vs.* *glo-1* Control
1	wild-type N2	94	11.32±0.43	82	11.08±0.67	98	n.s.		*glo-1(zu391)*	91	10.29±0.46	91	n.s.	85	10.12±0.58	98	n.s.
2	wild-type N2	103	9.38±0.36	101	8.92±0.53	95	n.s.		*glo-1(zu391)*	97	9.00±0.38	96	n.s.	94	10.29±0.33	114	<0.05
3	wild-type N2	106	8.64±0.36	90	9.52±0.53	110	n.s.		*glo-1(zu391)*	100	7.70±0.25	89	<0.05	75	8.50±0.38	110	n.s.
4	wild-type N2	91	7.74±0.34	73	8.05±0.66	104	n.s.		*glo-1(zu391)*	85	8.68±0.61	112	p=0.01	88	7.12±0.37	82	<0.05
**Overall**	**wild-type N2**	**394**	**9.27±1.52**	**346**	**9.39±1.28**	**101**	**n.s.**		***glo-1(zu391)***	**373**	**8.92±1.07**	**96**	**n.s.**	**342**	**9.01±1.50**	**101**	**n.s.**

Fig. S4a AA preconditioning

		Control		AA PC						Control				AA PC			
Assay No.	Genotype	n	Mean survival (hours)	n	Mean survival (hours)	% change *vs.* N2 Control	P value *vs.* N2 Control		Genotype	n	Mean survival (hours)	% change *vs.* N2 Control	P value *vs.* N2 Control	n	Mean survival (hours)	% change *vs.* *glo-1* Control	p value *vs.* *glo-1* Control
1	wild-type N2	76	9.18±0.52	80	9.60±0.69	105	n.s.		*glo-1(zu391)*	76	8.73±0.58	95	n.s.	85	8.28±0.40	95	n.s.
2	wild-type N2	92	10.15±0.55	94	9.43±0.34	93	p=0.072		*glo-1(zu391)*	85	8.84±0.42	87	<0.05	88	9.35±0.38	106	n.s.
**Overall**	**wild-type N2**	**168**	**9.67±0.68**	**174**	**9.51±0.12**	**98**	**n.s.**		***glo-1(zu391)***	**161**	**8.78±0.08**	**91**	**n.s.**	**173**	**8.81±0.75**	**100**	**n.s.**

Supplementary Table S2. PA14 pathogen assays

Fig. 8a-b ***glo-1*** **pathogen assays**

		Control		BA PC						Control				BA PC		
**Assay No.**	**Genotype**	**n**	**Mean survival (hours)**	**n**	**Mean survival (hours)**	**% change *vs.* N2 Control**	**P value *vs.* N2 Control**	**Genotype**	**n**	**Mean survival (hours)**	**% change *vs.* N2 Control**	**P value *vs.* N2 Control**	**n**	**Mean survival (hours)**	**% change *vs.* *glo-1* Control**	**P value *vs.* *glo-1* Control**
1	wild-type N2	77	44.42±1.86	102	52.94±1.57	119	p=0.01	*glo-1(zu391)*	121	38.17±1.36	86	<0.05	110	42.33±1.65	111	<0.05
2	wild-type N2	80	45.93±2.42	92	50.96±2.33	111	p=0.066	*glo-1(zu391)*	89	44.41±2.16	97	n.s.	78	45.26±3.11	102	n.s.
3	wild-type N2	95	43.71±1.65	109	48.34±1.88	111	<0.05	*glo-1(zu391)*	94	40.33±1.16	92	n.s.	89	43.01±1.60	107	n.s.
4	wild-type N2	98	40.00±1.49	112	52.77±1.58	132	<0.001	*glo-1(zu391)*	100	38.58±1.56	96	n.s.	115	42.33±1.65	110	p=0.058
**Overall**	**wild-type N2**	**350**	**43.51±2.52**	**415**	**51.25±2.14**	**117**	**<0.001**	***glo-1(zu391)***	**404**	**40.37±2.85**	**93**	**n.s.**	**392**	**43.23±1.39**	**107**	**n.s.**

Fig. 8c-d ***pgp-2*** **pathogen assays**

		Control		BA PC						Control				BA PC		
**Assay No.**	**Genotype**	**n**	**Mean survival (hours)**	**n**	**Mean survival (hours)**	**% change *vs.* N2 Control**	**P value *vs.* N2 Control**	**Genotype**	**n**	**Mean survival (hours)**	**% change *vs.* N2 Control**	**P value *vs.* N2 Control**	**n**	**Mean survival (hours)**	**% change *vs.* *pgp-2* Control**	**P value *vs.* *pgp-2* Control**
1	wild-type N2	77	44.42±1.86	102	52.94±1.57	119	p=0.01	*pgp-2(kx48)*	96	38.83±1.51	87	<0.05	96	38.67±1.68	100	n.s.
2	wild-type N2	80	45.93±2.42	92	50.96±2.33	111	p=0.066	*pgp-2(kx48)*	82	39.54±2.14	86	<0.01	92	37.76±1.63	95	n.s.
3	wild-type N2	95	43.71±1.65	109	48.34±1.88	111	<0.05	*pgp-2(kx48)*	98	35.18±1.41	80	<0.001	93	33.25±1.35	95	n.s.
4	wild-type N2	87	39.24±1.80	95	44.39±1.58	113	p=0.072	*pgp-2(kx48)*	99	33.53±1.39	85	<0.01	106	37.42±1.69	112	<0.05
**Overall**	**wild-type N2**	**339**	**43.33±2.88**	**398**	**49.16±3.69**	**114**	**<0.05**	***pgp-2(kx48)***	**375**	**36.77±2.88**	**84**	**<0.01**	**387**	**36.77±2.41**	**100**	**n.s.**

Supplementary Table S3. List of primer sequences

Gene	Sequence Name	Forward primer	Reverse primer
pgp-2	*C34G6.4a.1*	5'-TAA CGG CTA GAC TCA CGG AC-3'	5'-CCA GAG AGC ACA ATC AAC GG-3'
mrp-4	*F21G4.2.1*	5'-CGT TGG AAG TGA GCG ATC TG-3'	5'-CGG CGT GGT GTC ATT AGA AG-3'
K09C4.5	*K09C4.5.1*	5'-CGT CAC GAG CGA TTG AGT TT-3'	5'-TGA ATC CGG CAG TTT TGT GG-3'
glo-3	*F59F5.2a.1*	5'-CTT GGA GAA GTG CCC TTT CG-3'	5'-AAC GGC TTT GAC TGA GTT CG-3'
apb-3	*R11A5.1a.1*	5'-CTC GGT AGT GCT GTT TAC GC-3'	5'-GAG GAC GAG CTG AGT TGA GA-3'
vps-41	*F32A6.3a.1*	5'-AAT GCA AGG AGG AGT GGG AA-3'	5'-GCT GAT TTG TTG AAT GCG CC-3'
hsp-6	*C37H5.8.1*	5'-GAG ATC GTG GAA CCG GAA AGG A-3'	5'-CGG CAT TCT TTT CGG CTT CCT T-3'
hsp-60	*Y22D7AL.5a.1*	5'-GGA TAT TGT GCC GGC TCT TG-3'	5'-GCG TGT TCT TGC GGT TAT CT-3'
hsp-16.2	*Y46H3A.3a.1*	5'-CTT TAC CAC TAT TTC CGC CAG CTC-3'	5'-CTG TGA GAC GTT GAG ATT GAT GGC-3'
hsp-70	*C12C8.1.1*	5'-CGG TAT TTA TCA AAA TGG AAG GTT-3'	5'-TAC GAG CGG CTT GTC TTT T-3'
clec-60	*ZK666.6.1*	5'-CAC AAC TCC GTC TCC TTC CT-3'	5'-CCG CAG CTT TGT TGT AGG TT-3'
irg-1	*C07G3.2.1*	5'-TGA AAC TTG TGG AGG CCT CA-3'	5'- TGG CAT CTA GTT TCC AGG CT-3'
irg-5	*F35E12.5.1*	5'-TCT GCG GTG AAG AAG TAC GA-3'	5'-GTG GAA GCT TCT GTG TTG GG-3'
gst-4	*K08F4.7.1*	5'-GCA GAG GAA GAA GCT TAC GC-3'	5'-AGA AAT CAT CAC GGG CTG GT-3'
cyp-35B	*K07C6.4.1*	5'-GGT CAC GTC CAC TTC AGC TA-3'	5'-CGG GTA GAA CTT GTC GGG AT-3'
act-4	*M03F4.2a.1*	5 -ATC ACC GCT CTT GCC CCA TC-3	5'-GGC CGG ACT CGT CGT ATT CTT G-3
