# Supplementary material for: Lysosome-related organelles promote stress and immune responses in C. elegans
Source: Commun Biol. 2023 Sep 13;6:936. doi: 10.1038/s42003-023-05246-7 (PMC10499889; doi:10.1038/s42003-023-05246-7)
Supplement: Supplementary file 3 — Description of Additional Supplementary Data [file 42003_2023_5246_MOESM3_ESM.docx]

**Description of Additional Supplementary Files**

**File name:** Supplementary Data 1

**Description:** Source data presented in the main figures in Excel format.
